# Integrative approaches for predicting protein network perturbations through machine learning and structural characterization

**DOI:** 10.1016/j.jprot.2025.105439

**Published:** 2025-04-12

**Authors:** Bethany D. Bengs, Jules Nde, Sreejata Dutta, Yanming Li, Mihaela E. Sardiu

**Affiliations:** a Department of Biostatistics & Data Science, University of Kansas Medical Center, Kansas, USA; b Department of Cancer Biology, University of Kansas Medical Center, Kansas, USA; c University of Kansas Cancer Center, Kansas City, USA; d Kansas Institute for Precision Medicine, University of Kansas Medical Center, Kansas, USA

**Keywords:** Machine learning, Perturbation networks, Chromatin remodeling, Statistics

## Abstract

Chromatin remodeling complexes, such as the *Saccharomyces cerevisiae* INO80 complex, exemplify how dynamic protein interaction networks govern cellular function through a balance of conserved structural modules and context-dependent functional partnerships, as revealed by integrative machine learning and structural mapping approaches. In this study, we explored the INO80 complex using machine learning to predict network changes caused by genetic deletions. Tree-based models outperformed linear approaches, highlighting non-linear relationships within the interaction network. Feature selection identified key INO80 components (e.g., Arp5, Arp8) and cross-compartment features from other remodeling complexes like SWR1 and NuA4, emphasizing shared functional pathways. Perturbation patterns aligned with biological modules, particularly those linked to telomere maintenance and aging, underscoring the functional coherence of these networks. Structural mapping revealed that not all interactions are predictable through proximity alone, particularly with Arp5 and Yta7. By combining structural insights with machine learning, we enhanced predictions of genetic perturbation effects, providing a template for analyzing cross-species homologs (e.g., human INO80) and their disease-associated variants. This integrative approach bridges the gap between static structural data and dynamic functional networks, offering a pathway to disentangle conserved mechanisms from context-dependent adaptations in chromatin biology.

**Significance:**

By leveraging an innovative, integrative machine learning approach, we have successfully predicted and analyzed perturbations in the INO80 network with good accuracy and depth. Our novel combination of machine learning, perturbation analysis, and structural investigation approach has provided crucial insights into the complex’s structure-function relationships, shedding new light on its pivotal roles in affected pathways such as telomere maintenance. Our findings not only enhance our understanding of the INO80 complex but also establish a powerful framework for future studies in chromatin biology and beyond. This work represents a step forward in our understanding of chromatin remodeling complexes and their diverse cellular functions, laying the groundwork for future studies that can further refine our computational approaches and experimental techniques in this field.

## 1. Introduction

In recent years, the study of protein interactions within cells has gained considerable attention due to their essential roles in cellular processes. Proteins rarely function in isolation; instead, they engage in diverse interactions, from transient contacts to stable assemblies. These interactions can form intricate and dynamic networks that influence critical biological processes [1,2]. Among these, direct protein-protein interactions and modular relationships within protein complexes have been a primary focus for understanding their impact on the stability of interaction networks. Studies employing topological and network-based approaches [3–6] have highlighted the importance of interactions directly between proteins and within modules for maintaining structural integrity and influencing the functionality of complexes.

A notable example of such a network is the INO80 chromatin remodeling complex, a conserved protein complex with significant roles in nuclear processes such as transcription, DNA replication, and repair [7–9]. In *Saccharomyces cerevisiae*, INO80 is particularly crucial for maintaining genome stability [10]. Extensive structural characterization has provided insight into its organization, revealing distinct modules, including the core ATPase Ino80, the Rvb1/Rvb2 heterododecamer, and the Arp5-Ies6 module [11–14]. Additionally, topological data analysis has refined our understanding of interaction strengths within these modules, identifying key structural relationships that influence INO80’s stability and function [15,16].

Another key approach in recent research for studying protein complex dynamics is network perturbation. Perturbation of protein interaction networks can be achieved through biochemical or genetic approaches, including inhibitors, genetic mutations, and genetic deletion [15,17–20]. In *S. cerevisiae*, genetic deletion has been proven to be particularly effective in studying protein complex dynamics [21–25]. However, while this technique has significantly contributed to the characterization of network structures, there remains a gap in understanding how structural changes within protein complexes affect their functionality. Given the critical roles of protein interactions in cellular processes, this knowledge is essential for understanding the impact of these networks on organismal health.

To bridge this gap, integrative machine learning techniques offer promising solutions by enabling predictive modeling of network perturbation outcomes. Analyzing protein networks involves examining interactions among numerous proteins, resulting in complex and high-dimensional data. Techniques such as feature selection [26] and dimensionality reduction [27] play a crucial role in managing this complexity by pinpointing the most pertinent features, including specific proteins or interactions, within the network. Furthermore, rare events, such as mutations that lead to disease, adverse drug reactions, or unusual phenotypic responses, are challenging to predict due to their infrequency and the high dimensionality of biological data [28,29]. Machine learning models, particularly those designed for anomaly detection or rare event prediction, can be trained to recognize the subtle signals associated with these events [30].

Ensemble learning techniques can be employed to enhance the performance of these models. These methods can integrate both supervised and unsupervised machine learning approaches, leveraging their strengths to improve prediction accuracy and model interpretability [31–33]. Supervised learning techniques are particularly effective for predicting rare events, such as specific protein interactions, by learning from labeled data [30,34]. In contrast, unsupervised learning methods excel in discovering hidden patterns and structures within unlabeled data, making them valuable for exploratory analysis [34,35]. Here, we will use both techniques to analyze the dynamics of protein complex networks. In their recent work, Dutta et al. [36] propose an integrative machine learning approach employing twelve supervised machine learning methods to simultaneously allow for the prediction of rare events and feature structure selection. This approach not only enhances the accuracy of predicting rare events but also improves model interpretability through the incorporation of feature structures [36]. Thus, it is well-suited for the complexity and high dimensionality of omics data and the dynamic nature of biological networks, particularly when dealing with perturbed networks.

In this study, we leverage perturbation network analysis in conjunction with an integrative machine learning approach to predict the outcomes of perturbing the subunits of the INO80 complex in *S. cerevisiae*. By combining advanced statistical methodologies with machine learning techniques, we comprehensively analyze how genetic perturbations influence protein networks. First, we apply the statistical framework QPROT for differential expression analysis to quantify changes in protein abundance between wild-type and perturbed networks. We also explore the functional pathways associated with proteins exhibiting significant changes. We then incorporate structural data to provide mechanistic explanations for the observed genetic perturbations between specific subunit interactions within the INO80 complex. Finally, we utilize a machine learning approach that integrates supervised learning techniques with feature selection to predict network perturbations within the complex while considering its biological structure. Our findings significantly enhance the understanding of protein network dynamics and underscore the transformative potential of integrating network perturbation analysis with structural data and machine learning techniques in areas such as cross-species analysis and disease research.

## 2. Methods

### 2.1. Input datasets

We utilized two publicly available affinity purification-mass spectrometry datasets [3,16], which consist of the total number of spectra for proteins purified from wild-type and genetically perturbed subunits of four chromatin remodeler complexes in *S. cerevisiae*. The yeast INO80 complex comprises fifteen subunits, including ten conserved across yeast and humans and five specific to yeast. In the wild-type dataset, we considered purifications of seven of the ten conserved INO80 subunits. We also included purifications of eight subunits from three other chromatin remodeler families in yeast (i.e SWi/SNF, NUA4 and SWR-C) to validate the accuracy of machine learning prediction. Our analysis incorporated replicates, resulting in thirty-four purifications (see Supporting Information, Table S1, for a complete list of purifications, including the purified subunits and replicate information).

For the perturbation dataset, we analyzed purifications of INO80 components when individual subunits were genetically deleted from the complex (see Supporting Information, Figure S1, for a visualization of the genetic deletion process). We analyzed eleven purifications via four TAP-tagged subunits—Ino80, Arp8, Ies2, and Ies6—when six different subunits were genetically deleted from the complex. These deletion strains were *Ino80-TAP ies4Δ*, *Ino80-TAP arp8Δ*, *Ino80-TAP arp5Δ*, *Ino80-TAP ies2Δ*, *Ino80-TAP ies5Δ*, *Ino80-TAP nhp10Δ*, *Arp8-TAP arp5Δ*, *Arp8-TAP ies2Δ*, *Ies2-TAP arp8Δ*, *Ies2-TAP ies4Δ*, and *Ies6-TAP ies4Δ*. The six subunits were chosen strategically based on the modularity of the INO80 complex to ensure a diverse representation of its functional components. The INO80 complex is comprised of five distinct modules: the Arp5 module, the Arp8 module, the Nhp10 module, the Ino80/Ies2 module, and the Rvb1/Rvb2 module. We selected deletion strains spanning different modules to capture a diverse range of interactions, enabling a broader assessment of how perturbations affect the complex’s composition and function. Twenty-five purifications were analyzed, accounting for replicates across the experiments (see Supporting Information, Table S2, for a complete list of purifications, including the purified deletion strains and replicate information).

The datasets are represented in a bait–prey matrix: each column corresponds to the purification of a subunit of a chromatin remodeler complex (the bait protein), and each row corresponds to an interacting protein (the prey protein). The matrix cell values represent each protein’s distributed spectral (dS) counts, reflecting the total number of spectra detected for each protein. When combining the datasets, a total of 2128 prey proteins were identified in both the wild-type and perturbation datasets. For our analysis, we employed seven distinct normalization methods (log scaling, TopS [3], min-max, standardization, arcsine, percentage row, and no normalization) in combination with our machine learning models to identify the best-performing approach.

### 2.2. Differential protein expression analysis

We calculated the fold changes between the purifications of the INO80 subunit in the wild-type network and the INO80 TAP-tagged subunits in the perturbed network using the QPROT statistical framework (version 1.3.5. QPROT). QPROT implements a Bayesian hierarchical model for differential protein expression analysis, allowing for detecting and quantifying changes in protein abundance between the networks [37]. The proteins’ spectral counts served as input data for QPROT. We used *Z*-scores and false discovery rates (FDRs) derived from QPROT to assess the significance of the differences between the networks. These metrics also served as criteria for one of our two machine learning outcomes in the analysis. We considered proteins with a *Z*-score greater than or equal to 1.5 or less than or equal to – 1.5 and an FDR less than or equal to 0.05 in at least one perturbed network significant for further analysis.

To visualize patterns in these significant proteins, we employed hierarchical clustering analysis to generate a heatmap using ClustVis, a web-based tool for clustering visualization [38]. The *Z*-score values obtained from QPROT were used as input to identify clusters based on similarity in protein abundance changes. Additionally, upset plots were constructed using the UpSetR package in R, specifically utilizing the ‘upset’ function, to visualize similarity across the perturbed networks [39]. These plots depict overlaps and unique occurrences of proteins in the perturbed networks based on their presence or absence in the networks and their significance scores derived from QPROT.

### 2.3. t-SNE and pathway analysis

To explore the similarities among proteins in the INO80 complex, we applied t-SNE analysis followed by k-means clustering to the significant proteins based on the *Z*-scores from the differential protein expression analysis using QRPOT. The optimal number of clusters for our data was determined using the ‘clusGap’ function in the cluster package [40] and the ‘NbClust’ function in the NbClust package [41] in R, which consistently identified the optimal number of clusters as k = 3. We used t-distributed stochastic neighbor embedding (t-SNE) to visualize the clusters. All computations were conducted in R, and we employed the ‘kmeans’ function for cluster partitioning and the ‘daisy’ function to calculate pairwise dissimilarities based on Euclidean distances between observations in the dataset.

We used ConsensusPathDB-yeast to investigate the biological pathways associated with the proteins in each cluster. ConsensusPathDB is a meta-database that integrates interaction network data for three organisms (human, mouse and yeast) to facilitate the identification of subnetworks and biological functions associated with lists of molecules [42]. Among its analytical tools, ConsensusPathDB employs over-representation analysis (ORA) to assess whether the proportion of genes or proteins in a list is present in a pathway more frequently than would be expected from random chance [43,44]. Using this method, we identified each cluster’s top five to ten enriched Reactome pathways. Statistical significance was calculated using Fisher’s exact test and measured by *P*-values.

### 2.4. Structural analysis of protein-protein interactions

We used genetic interaction data to identify functionally relevant protein pairs within chromatin remodeling complexes. Unlike direct physical interactions or membership within the same complex, genetic interactions highlight functional dependencies between genes. To this end, we selected the two protein pairs representing distinct modules within the INO80 complex: the Arp8/Hir3 and Arp5/Yta7 interactions. Hir3 and Yta7 were randomly selected from the pool of proteins that were both absent in at least one module of the complex and exhibited significant differential expression (see Supporting Information, Table S2, last column colored in red). This selection indicated their potential significance regarding protein perturbations within chromatin remodeling complexes. For instance, a genome-wide screen for *S. cerevisiae* deletion mutants affecting telomere length identified Yta7 as one of the genes involved in telomere function [45].

We employed the AlphaFold protein structure prediction system [46] to generate high-confidence 3D structures of the Arp8/Hir3 and Arp5/Yta7 protein complexes. We retrieved the sequences of amino acids of the individual protein from UniProt, which we then submitted to the AlphaFold server for the structure prediction while using the default settings of AlphaFold. To analyze the protein-protein interactions in the predicted complexes, we generated contact maps using customized scripts. Contact maps provide detailed visualizations of atomic interactions between the residues of the interacting proteins, which makes them effective for identifying the specific regions of protein interfaces that contribute to the stability of protein complexes. These maps were generated by calculating pairwise atomic distances (typically <30 Å) between residues in the predicted 3D structures of the Arp8/Hir3 and Arp5/Yta7 complexes. Residues that fall within this distance threshold were considered to be in contact.

### 2.5. Machine learning

We employed the integrative machine learning approach developed by Dutta et al. [36] to predict the outcome of genetically perturbing the INO80 complex. This method integrates twelve supervised machine learning methods in combination with seven distinct normalization methods to thoroughly evaluate and identify the best performing models for predicting rare events within a dataset. It also implements both the ‘varImp’ function in the R package caret and stepwise AUC algorithm to select the most important features in the prediction of these events [36,47]. The output is a persistent biomarker structure that categorizes features into persistently selected, persistently fluctuating, and persistently unselected based on their predictive importance.

For our analysis, we used the distributed spectral (dS) counts of proteins as input. To optimize model performance, the input for the first outcome was normalized using TopS [3], while percentage row normalization was applied to the input for the second outcome. Model performance was assessed using five evaluation metrics: accuracy, sensitivity, specificity, area under the receiver operating characteristic curve (ROC-AUC), and Cohen’s Kappa. We validated our results by applying Synthetic Minority Over-sampling Technique (SMOTE) to address class imbalance and ensure robust evaluation.

We evaluated the machine learning models for two distinct binary outcomes. The first outcome is determined by the presence or absence of prey proteins in the perturbed networks, as observed from the total spectral counts. For this outcome, we assigned one (1) to proteins absent in at least one module of the perturbed INO80 complex, while we assigned zero (0) to proteins present in all the perturbed networks. The perturbation dataset was derived from six genetically perturbed subunits of the INO80 complex, representing four of its five modules. However, prior research suggests that we can condense these modules into three modular domains: the Nhp10 module, the Arp8 module, and the Ino80 core, which encompasses the Arp5, Ino80/Ies2, and Rvb1/Rvb2 modules [48]. Based on this framework, we grouped the six perturbed subunits into three domain-representative pairs. Arp8 and Ies4 correspond to the Arp8 module, Ies5 and Nhp10 to the Nhp10 module, and Arp5 and Ies2 to the Ino80 core. A protein was considered absent in a module if it was absent in the networks of both subunits associated with that module. The second outcome is defined by the significance of proteins based on *Z*-score and FDR combinations obtained from QPROT. Proteins with a significant Z-score and FDR in at least one of the perturbed networks were assigned one (1), while proteins not significant across all perturbed networks were assigned zero (0).

**Fig. 1.**
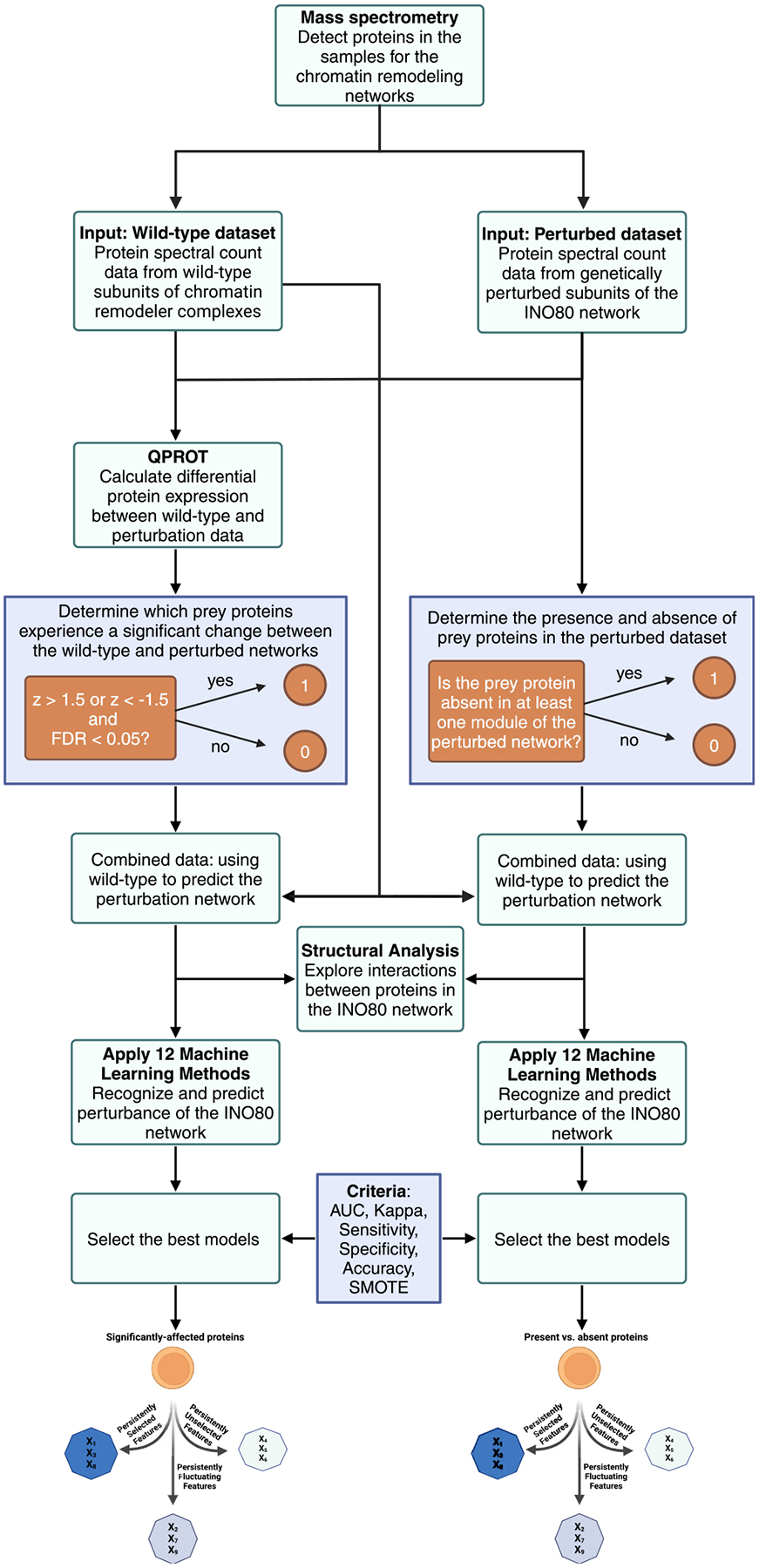
Workflow for predicting the perturbation of protein interaction networks using an integrative machine learning approach.

The summary of the methodology employed in this study is illustrated in the workflow in Fig. 1. This workflow visually represents the sequential steps followed throughout the research process—from data preparation and statistical analysis to machine learning modeling and interpretation—to provide a clear and comprehensive overview of our methodology.

## 3. Results

### 3.1. Identifying altered proteins in the INO80 network

**Fig. 2.**
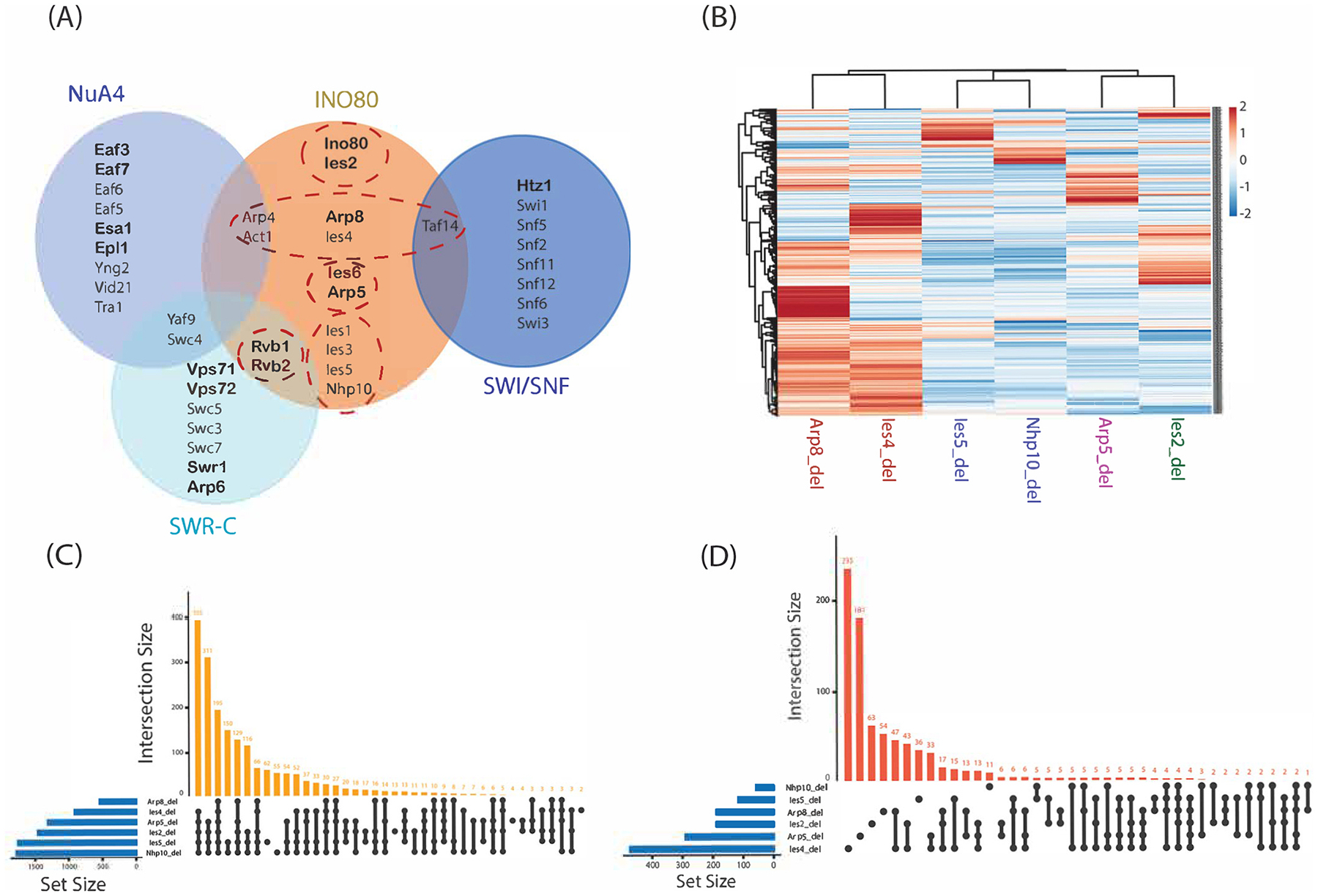
Clustering of the differential protein expression of the INO80 TAP-tagged subunits in the perturbed INO80 network. (A) The diagram illustrates the INO80 complex and three other chromatin remodeling complexes used as input for the machine learning models. The input bait proteins are highlighted in bold, and the subunits of the INO80 complex are grouped into modules, indicated by red dashed circles. The deletion subunits were selected from four of the five modules. (B) The hierarchical clustering of the six analyses is shown, where the quantitative *Z*-scores from QPROT were used as input. Only proteins with *Z*-scores of Z > 1.5 or Z < −1.5 and FDR < 0.05 in at least one of the six deletion strains were included in the clustering. The color intensity represents the Z-score values, where red indicates high Z-scores, and blue indicates low Z-scores. (C, D) Upset plots were created to represent the similarity between the deletion strains based on (C) the presence or absence of prey proteins in the perturbed networks and (D) the significantly affected proteins in the perturbed networks (z > 1.5 or z < −1.5 and FDR < 0.05). The value above each bar represents the number of intersected proteins affected by multiple deletions, while the connected dots below each bar denote the specific combination of deletion strains contributing to each intersection.

Our comprehensive protein network includes data from the wild-type of four chromatin remodeler families in yeast—INO80, SWI/SNF, NUA4, and SWR-C—and perturbed states of the INO80 complex (see Supporting Information, Tables S1 and S2). Fig. 2A provides an overview of the four remodeler complexes analyzed in this study, highlighting in bold the subunits used as bait proteins in the wild-type dataset. These data offer a detailed insight into the dynamics and interactions within these chromatin remodeler families, shedding light on their roles in gene regulation and chromatin structure maintenance. We conducted a thorough analysis of differential protein expression to investigate the intricate topology of the perturbation network within the INO80 complex. This involved comparing the wild-type network with genetically perturbed networks of the INO80 complex in *S. cerevisiae*. We focused on six Ino80 TAP-tagged deletion strains: *Ino80-TAP ies4Δ*, *Ino80-TAP arp8Δ*, *Ino80-TAP arp5Δ*, *Ino80-TAP ies2Δ*, *Ino80-TAP ies5Δ*, and *Ino80-TAP nhp10Δ*. Using QPROT, we calculated the fold change between the wild-type affinity-purified INO80 subunit and each deletion strain. Out of 2128 proteins, 872 experienced a significant change in spectral counts between the wild-type and perturbed networks in at least one deletion strain (Z > 1.5 and FDR < 0.05, or Z < – 1.5 and FDR < 0.05).

We applied hierarchical clustering to the data from these six deletion strains, focusing on proteins identified as significant from our differential protein expression analysis (Fig. 2B). The clustering revealed four distinct modular patterns, aligning with the modularity outlined in the INO80 complex shown in Fig. 2A. Specifically, deletions of arp8 or ies4 from the INO80 complex led to similar changes in protein abundance, reflecting their co-localization within the same module of the INO80 complex. Similarly, deletions of ies5 or nhp10 exhibited comparable fold changes in protein abundance between the networks, consistent with their presence in the same complex modules, whereas arp5 and ies2 deletions showed distinct fold change patterns. These results confirm the modular organization of the INO80 complex in *S. cerevisiae* and suggest that this modularity plays a role in predicting the impact of perturbations.

We also used upset plots to visualize disturbed proteins’ overlap and unique occurrences across the perturbed networks (Fig. 2C and D). The first plot shows the presence and absence of proteins across the six perturbed networks, where each column represents a deletion strain, and the bars represent the number of proteins absent in one or more strains. We observed a substantial overlap among deletion strains, with 395 proteins absent across all strains except arp8 and 195 proteins absent across all strains. The second plot illustrates the distribution of proteins identified by QPROT across the deletion strains, where the bars represent proteins undergoing significant changes in abundance. In contrast to the first plot, this plot reveals a higher number of unique significant proteins for each strain. Notably, ies4 had 235 uniquely significant proteins, and arp5 had 181 proteins, indicating strain-specific responses to perturbation within the INO80 network.

### 3.2. Similarity of altered proteins in the INO80 network

**Fig. 3.**
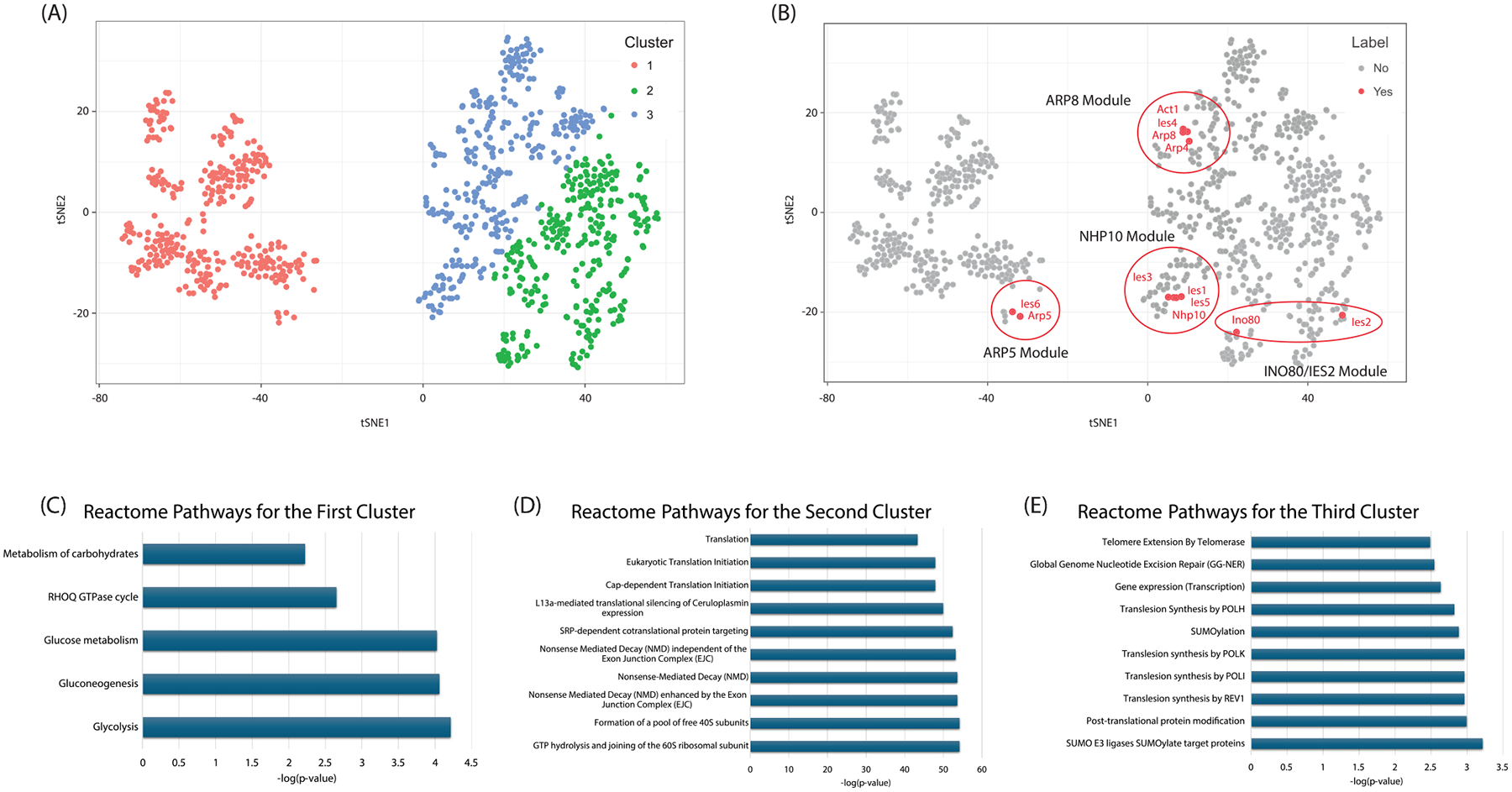
Pathway analysis of the prey proteins clustered by the differential protein expression of the perturbed INO80 bait proteins. (A) tSNE was used to visualize the quantitative Z-scores of the prey proteins from QPROT. Here, the data points represent only the proteins with Z-scores of Z > 1.5 or Z < −1.5 and FDR < 0.05. Three clusters of the prey proteins were found and are visualized in the tSNE plot, where the red points correspond to the first cluster, the green points to the second cluster, and the blue points to the third cluster. (B) The location of the INO80 components within the clusters. Eight of the twelve subunits are found within the third cluster. (C, E) Pathway analysis was performed for each of the three clusters of prey proteins. The top five to ten enriched classes of Reactome pathways are shown for (C) the first cluster (red points in the tSNE points from part A), (D) the second cluster (green points in the tSNE plot from part A), and (E) the third cluster (blue points in the tSNE plot from part A).

Next, we sought to explore the similarities among proteins in the INO80 complex using k-means clustering on the *Z*-scores of significant proteins from QPROT (Fig. 3A and B). When partitioning the data, we found three distinct clusters of proteins. Notably, several INO80 complex subunits were grouped in the third cluster, with others distributed across different clusters. This grouping reflects the modular nature of the complex, as proteins within the same module exhibited relatively small pairwise distances. Arp8, Ies4, Arp4, and Act1 were closely grouped in the third cluster, indicating strong correlations among these subunits. Similarly, Ies5, Ies3, Ies1, and Nhp10 formed another tightly clustered group. In the first cluster, Ies6 and Arp5, which belong to the same module, were grouped together. Ino80 and Ies2, part of another module, appeared together in the second cluster, though their pairwise distance was larger compared to other subunit groupings. This clustering pattern underscores the structural organization and interrelations of subunit modules within the INO80 complex.

To examine the functional roles of proteins within these clusters that may be altered by the perturbation approach, an over-representation analysis (ORA) was conducted using ConsensusPathDB-yeast to discern associated biological pathways. We focused on the top five to ten enriched Reactome pathways, using the negative logarithms of the *P*-values obtained from ORA (Fig. 3C through 3E). Our results revealed that the predominant pathways across all clusters were primarily linked to metabolic processes and DNA repair, aligning with known functions of the INO80 complex. Interestingly, the third cluster, which contains a majority of the INO80 components, showed enrichment in pathways related to telomere maintenance. This finding reinforces previous research on the role of the INO80 complex in maintaining telomere structure and function [49].

### 3.3. Structural analysis of protein-protein interactions within the INO80 network

To further provide mechanistic explanations for the observed genetic perturbations of chromatin-associated protein complexes, we conducted a structural analysis of protein-protein interactions involving key subunits within the network. This analysis complements the deletion experiments and our machine learning approach to predict perturbed proteins, offering a comprehensive view of the intricate relationships between these components. By integrating these findings with the observed enrichment in metabolic and telomere-related pathways, we gain a deeper understanding of how perturbation influences both structural integrity and functional outcomes of the INO80 complex.

**Fig. 4.**
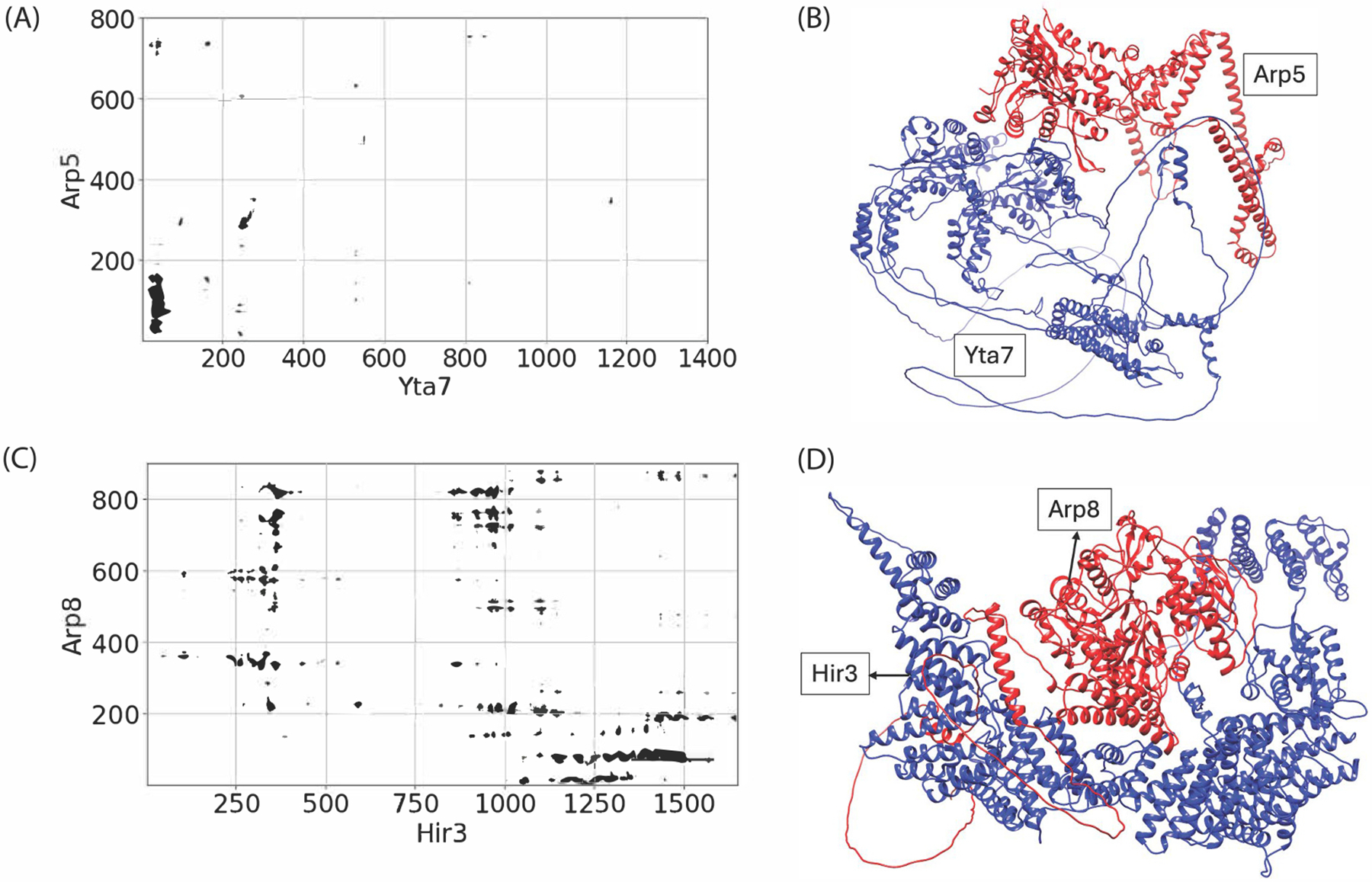
Structural analysis of protein-protein interactions between (A, B) Arp5 and Yta7 and (C, D) Arp8 and Hir3. The contact maps in (A) and (C) display the interacting residues, with the axes representing specific residue positions of the proteins involved. In (B) and (D), the 3D protein structures highlight the spatial organization of Yta7 and Hir3 in relation to Arp5 and Arp8, respectively. Interacting residues are visually distinguished to emphasize key interaction sites.

Fig. 4 illustrates these interactions, focusing on the relationships between Arp5 and Yta7 (Fig. 4A and B) and between Arp8 and Hir3 (Fig. 4C and D). The contact map and 3D visualization for Arp5 and Yta7 (Fig. 4A and B) revealed minimal direct contact between these two proteins, with interaction points sparsely distributed. Notably, the limited contact observed is primarily concentrated around the N-terminal regions of both proteins. This sparse structural interaction contrasts with our deletion experiments, which showed significant perturbation effects when Arp5 was removed. This discrepancy highlights the complex nature of protein interactions within chromatin remodeling complexes. It suggests that indirect effects or other cellular mechanisms may play a crucial role in the functional relationship between Arp5 and Yta7. Interestingly, recent cryo-EM analysis has shown that Yta7 assembles into a three-tiered hexamer with a unique bromodomain tier that functions in nucleosome disassembly [50]. Combined with our observations, this structural insight suggests that the functional interaction between Arp5 and Yta7 may be mediated through higher-order chromatin structures or other complex components, rather than direct protein-protein contacts.

In contrast, the contact map and 3D visualization for Arp8 and Hir3 (Fig. 4C and D) showed moderate interaction between the proteins, with contact points distributed more consistently across the sequence. The denser interaction points—particularly near the N-terminal region of Arp8 and the C-terminal region of Hir3—suggest a direct contact between Arp8 and Hir3. This more substantial interaction aligns with our predictions of the higher likelihood of perturbation effects when these proteins were targeted. Structural analysis of Arp8 reveals several insertions in the conserved actin fold that explain its inability to polymerize [51]. Most notably, one insertion wraps over the active site cleft, potentially rigidifying the domain architecture while maintaining features shared with actin. This structural arrangement suggests an allosterically controlled ATPase activity, which may be crucial for Arp8’s function in chromatin remodeling complexes. Furthermore, quantitative binding studies have shown that Arp8 and the Arp8-Arp4-actin-HSA sub-complex of INO80 strongly prefer nucleosomes and H3-H4 tetramers over H2A-H2B dimers [51]. This preference suggests that Arp8 functions as a nucleosome recognition module within the INO80 complex, potentially explaining its functional importance in our deletion experiments.

These structural insights, combined with our deletion experiments and machine learning predictions, provide a multi-faceted view of protein interactions and their functional implications within chromatin remodeling complexes. They highlight the complex interplay between protein structure, interaction patterns, and the broader effects of protein perturbations within cellular networks. Our findings underscore the importance of integrating structural, genetic, and functional data to fully understand the roles of these proteins in chromatin dynamics and gene regulation.

### 3.4. Machine learning for prediction

We used an integrative machine learning approach to predict the outcomes of perturbing the INO80 complex and to identify the most important features in this prediction. For our analysis, we considered two outcomes: the first is based on the presence and absence of proteins in the perturbed networks, and the second is based on the significance of fold changes of proteins between the wild-type and perturbed networks calculated using QPROT. Tables 1 and 2 summarize the results obtained for each outcome. The models were trained on the 2128 prey proteins identified in the wild-type and perturbed networks, with 80 % allocated to training and 20 % to testing. The model for the first outcome was evaluated using 5-fold cross-validation, while the model for the second outcome used 8-fold cross-validation to optimize the performance of individual machine learning models.

For the first outcome, identifying the presence or absence of proteins in the perturbed networks, the tree-based models such as Decision-Tree and XGBoost demonstrated the highest overall performance. The Decision Tree model achieved an accuracy of 82.0 %, sensitivity of 81.0 %, specificity of 87.0 %, and an area under the curve (AUC) of 84.0 %. XGBoost showed an accuracy of 83.0 %, a sensitivity of 82.0 %, a specificity of 85.0 %, and an AUC of 83.0 %. These metrics highlight the strong predictive capabilities of these models in identifying proteins that are either present or absent in at least one module of the INO80 complex in its perturbed networks.

To further assess model reliability, we calculated Cohen’s Kappa for the tree-based boosting models. The Decision Tree model achieved a kappa of 0.54, the Random Forest model achieved a kappa of 0.59, XGBoost achieved a kappa of 0.55, and ADABoost achieved a kappa of 0.56. These results indicate moderate to substantial agreement beyond chance, reinforcing the robustness of these models for predicting this outcome. Therefore, the tree-based methods provide the most intuitive display of the predictive details of the model.

To validate our models’ performance and address class imbalance, we applied SMOTE to ensure a more thorough evaluation. After balancing the data, we observed substantial improvements in model performance across many models. Notably, the tree-based models maintained strong performance, with kappa values around 0.60 for all except the Decision Tree model, which significantly declined in performance. This consistency underscores their stability and effectiveness in predicting the presence or absence of proteins in the perturbed networks.

For the second outcome, detecting significant changes in protein abundance from the wild-type to the perturbed networks, the tree-based boosting models such as XGBoost and ADABoost exhibited the best overall performance. XGBoost achieved an accuracy of 63.8 %, a sensitivity of 54.9 %, a specificity of 70.4 %, and an AUC of 62.7 %. ADABoost had an accuracy of 64.5 %, a sensitivity of 41.2 %, a specificity of 81.9 %, and an AUC of 61.6 %. Compared to the models for the first outcome, these models displayed less robust predictive capabilities for identifying proteins that undergo significant changes between the wild-type and perturbed networks.

Cohen’s Kappa values further supported this observation, with XGBoost achieving a kappa of 0.255 and ADABoost a kappa of 0.242. These lower values indicate weaker agreement beyond chance compared to the models for the first outcome, suggesting that while tree-based boosting models offer some predictive power, their reliability in this context is more limited.

After applying SMOTE to the dataset for the second outcome, we did not observe as substantial an improvement in model performance across algorithms as we did for the first outcome. However, the tree-based boosting models, particularly XGBoost and ADABoost, maintained their performance, with kappa values of 0.248 and 0.21 respectively. This further supports the stability of these models in detecting significant changes in protein abundance from the wild-type to the perturbed networks.

**Fig. 5.**
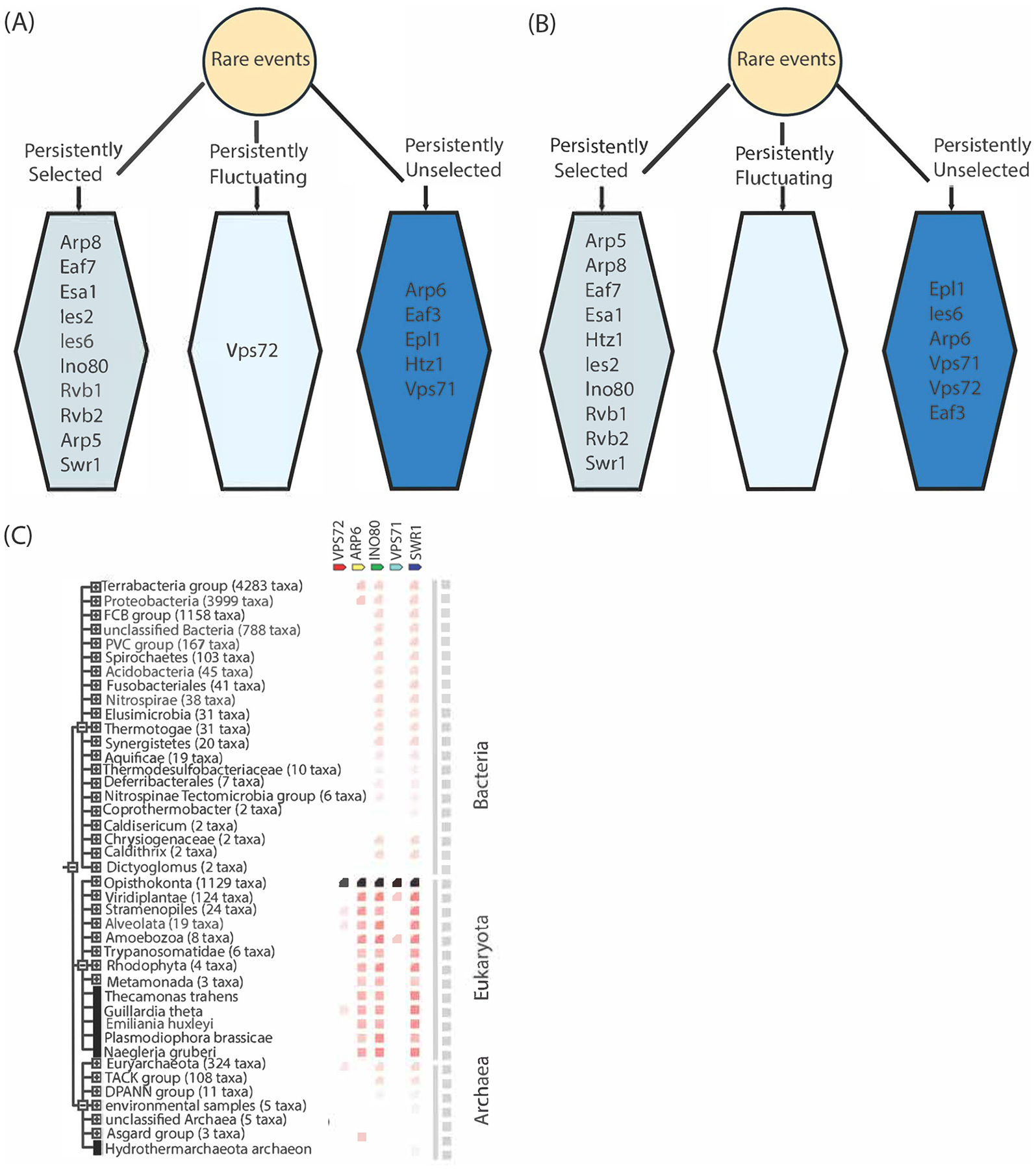
Prediction of perturbation patterns in the INO80 complex using integrative machine learning. (A, B) Persistent feature structures indicating subunits persistently selected, fluctuating, or unselected in machine learning models for (A) the outcome of protein presence or absence in the perturbed networks and (B) the outcome of significantly affected proteins. (C) Phylogenetic tree for the features from the INO80 and SWR complexes, illustrating the co-occurrence of selected subunits of the INO80 chromatin remodeling complexes. Gene families with similar occurrence patterns across genomes are shown. The color scale represents, for each gene of interest, the similarity of its best hit in a given STRING genome. Correlations of these presence/absence profiles can predict interactions. For groups of genomes that are collapsed in the phylogenetic tree, two distinct colors indicate the lowest and highest similarity observed within that clade. The Ino80 protein exhibits a higher similarity with Swr1 than with Vps71, Vps72, and Arp6.

We also illustrated the persistent biomarker structures to identify the most important subunits for predicting each outcome of perturbing the INO80 complex. For the first outcome, all seven subunits of the INO80 complex considered for this study were persistently selected predictors in the model compared to only two subunits from the other Remodeler complexes (Fig. 5A). This indicates that the IN080 complex subunits significantly contributed to the model’s predictive accuracy, thus supporting the biological context of our data. In contrast, for the second outcome, feature selection did not reveal as clear a distinction among the subunits of the various Remodeler complexes. Six INO80 complex subunits and four subunits from the other Remodeler complexes were persistently selected as features in the model, enriching the association of other remodelers with the INO80 complex (Fig. 5B).

In our analysis, we anticipated that there would be discernible variations in the outcomes of the machine learning process. This expectation stems from our use of quantitative values to assess the outcome. We observed that the proteins that undergo alterations are primarily associated with the complex, as opposed to the binary outcome. In the latter scenario, we observed a higher occurrence of altered proteins outside the complex. These observations underscore the complexity of protein interaction networks and the potential far-reaching effects of perturbations to chromatin remodeling complexes. They also emphasize the value of employing multiple analytical approaches to comprehensively understand the system dynamics. Furthermore, the Remodeler network is function-based, has a branched, interconnected topology, and shares many components. This complex biological model is, therefore, excellent to be studied with machine learning and different outcomes.

## 4. Discussion

In this study, we used an integrative machine learning approach to predict perturbation of the INO80 complex in *S. cerevisiae* to better understand the relationship between the structure and function of protein complexes. While previous studies have structurally characterized protein complexes like the INO80 complex, the intricate interactions within these systems present a significant challenge in understanding how their structural features affect functionality. Network perturbation analysis offers a valuable approach to studying these structure-function relationships by altering specific aspects of a protein complex. This, in turn, helps us better understand the roles of individual components and their contributions to the functionality of the complex. Our findings demonstrate the usefulness of perturbation analysis in understanding its functional mechanisms.

Exploratory analysis confirmed the modularity of the INO80 complex and provided some insight into its functionality. Hierarchical clustering identified three distinct patterns of protein abundance changes, aligning with previous topological studies of the INO80 complex. We observed that components within the same modules experienced similar changes in protein abundance, indicating that perturbation patterns are consistent within modules. This finding supports the potential use of modularity in protein complexes as a predictive tool for understanding network perturbations. This is further supported by our upset plots, which highlighted both shared and strain-specific perturbation patterns. Specifically, the first plot, visualizing the presence and absence of proteins in the perturbed networks, revealed a significant overlap among deletion strains. In contrast, the second plot, which visualized significant quantitative changes in protein abundance, highlighted strain-specific perturbations. These results suggest both the modular nature and strain-specific responses of the INO80 complex. The t-SNE approach and K-means clustering of the significant proteins also grouped all components of the INO80 complex by modularity, except for Ies2. This exception is not surprising, though, as Ies2 is a known outlier in modularity and has distinct roles from other components such as Ies6 and Arp5 [12,52]. Upon examining the pathways associated with these clusters, we discovered that altered proteins in the third cluster, encompassing many INO80 components, are involved in telomere maintenance pathways. Telomeres, which are regulated by subunits of the INO80 complex, are crucial for protecting chromosome ends and are linked to cellular aging and age-related diseases, including Alzheimer’s disease [53]. Given that previous research has suggested a link between the INO80 complex and Alzheimer’s disease, our findings support a potential connection between the proteins in the third cluster and aging processes [24,54]. Future research should focus on how perturbations of the INO80 complex affect aging by investigating perturbation patterns that impact its role in telomere regulation.

The structural complexity of protein complexes like INO80 reveals the intricate nature of protein interactions within cellular networks, challenging traditional understanding through nuanced interaction patterns. By analyzing interactions of Arp8 and Arp5—subunits from distinct modules within the complex—with Hir3 and Yta7—proteins that exhibited both significant differential expression and absence in at least one module of the complex—we explored the complex’s functionality from multiple structural perspectives. However, our investigation showed that protein interactions are not always predictable through direct structural mapping. The interaction between Arp5 and Yta7 showed minimal structural contact yet exhibited significant functional interdependence, while Arp8 and Hir3 displayed more consistent contact points, suggesting direct functional coordination. This complexity is particularly evident in telomere maintenance and chromatin dynamics, where Yta7 and Arp5 play crucial roles: Yta7’s bromodomain-like region and AAA ATPase domain contribute to chromatin structure, while Arp5, a key subunit of the INO80 chromatin remodeling complex, is directly implicated in telomere regulation [42,49]. The INO80 complex localizes preferentially to telomeres, promoting recombinational telomere maintenance by potentially altering chromatin structure [42]. The genetic deletion experiments used in this study suggest that Yta7 and INO80 may interact in parallel or cooperatively, reinforcing the importance of chromatin remodeling in telomere maintenance [50].

This observed functional synergy, despite minimal direct physical contact, demonstrates why our machine learning models prioritize functional metrics (such as chromatin remodeling efficiency) over structural proximity features when predicting complex-wide effects. Specifically, the models captured the indirect yet significant impact of Arp5-Yta7 cooperation on INO80 activity, demonstrating the importance of considering functional relationships that may not be immediately apparent from structural data alone. By integrating structural mapping, genetic perturbations, and machine learning, our approach reveals how the INO80 complex balances direct interactions and indirect functional coordination to regulate chromatin dynamics, underscoring the need for comprehensive methods to understand protein network behaviors.

Using this comprehensive outlook, we applied an integrative machine learning approach to bridge structural and functional insights of the INO80 network, achieving notable results in developing strong predictive models for perturbation combined with the biological structure of the complex. For the first outcome dataset, with TopS transformation [3], tree-based models showed superior accuracy, sensitivity, specificity, AUC, and kappa performance, while linear models and Support Vector Machines (SVM) exhibited poorer overall performances. One possible explanation is that tree-based models typically have non-linear decision boundaries, whereas models such as Naïve Bayes and LDA have linear decision boundaries. Furthermore, all models performed well in terms of accuracy, sensitivity, and AUC. The linear models and SVMs performed poorly only in specificity. This suggests that our data may be defined by complex, nonlinear relationships between the features that are better captured by the tree-based models [55].

The application of SMOTE to the first outcome dataset validated these results by demonstrating the robustness of the tree-based boosting models for this data. Our results correlate with previous studies, which shows that SMOTE performs well with XGBoost [56]. By generating synthetic samples for the minority class, SMOTE improved model performance across various algorithms, indicating that class imbalance was a significant issue in the original dataset [56,57]. This led to more reliable predictions for the presence and absence of proteins in perturbed networks. However, it is worth noting that tree-based boosting models performed well even without SMOTE, demonstrating their suitability for our dataset. Notably, the Decision Tree model’s performance deteriorated after applying SMOTE, suggesting that the introduction of synthetic samples may have hindered its ability to capture complex, non-linear relationships in the data [58]. This underscores the need for careful model selection and validation in our analysis.

The feature selection analysis of our integrative machine learning approach also supported the biological structure of the INO80 complex. The biomarker structure for the first outcome dataset revealed that components of the INO80 complex were the most persistently selected features compared to components from other Remodeler complexes. This is what we anticipated considering that INO80 complex components should be crucial for predicting perturbations within their complex. We also observed three components from other Remodeler complexes—Swr1, Eaf7, and Esa1—were consistently selected in our models. This is understandable, though, given the locations and roles of these subunits. Swr1 is the motor subunit of the SWR1 complex, which is closely related in structure and function to the INO80 complex [59]. This may make it exceptionally responsive to perturbations in the INO80 complex. Also, previous perturbation studies have indicated that Eaf7, a subunit of the NuA4 complex, exists independently as a trimer with Eaf3 and Eaf5, which is found on coding regions involved in active transcription [60,61]. Given the INO80 complex’s function in transcription, this association may cause Eaf7 to be more responsive to perturbations within the INO80 complex. Esa1, a histone acetyltransferase, is the catalytic subunit of the NuA4 complex that plays a crucial role in chromatin dynamics, particularly in the regulation of transcription and DNA repair [62,63]. Its function in acetylating histone H4 may be influenced by the chromatin remodeling activities of INO80, which may make it similarly sensitive to perturbations within the complex [63].

For the second outcome dataset, our results indicate that the tree-based models performed best with percentage row transformation, specifically XGBoost and ADABoost. This is similar to the first outcome dataset, suggesting that there may be a non-linear relationship between the variables, which tree-based models are better suited to capture. However, these models did not perform as strongly as those for the first outcome dataset. The discrepancy could be attributed to the data’s non-linearity and the lower percentage of rare events in the second outcome dataset (41 % versus 82 % in the first outcome dataset) [29]. Despite the reduced class imbalance for this dataset, the class boundaries may be less distinct because of the complexity and distribution of the outcomes [28], which could contribute to the poorer performance of the models for the second outcome dataset. The application of SMOTE confirms this, as balancing the data did not lead to significant improvements in predicting changes in protein abundance. However, this presents an opportunity to explore more advanced approaches. By integrating machine learning with domain-specific biological insights, we may enhance predictive performance in this complex biological context.

The feature selection analysis for the second outcome dataset also demonstrated weaker performance in the biological context of our research. Although most INO80 complex components were persistently selected for the machine learning models, Ies6 was persistently unselected. Additionally, we observed four components from other Remodeler complexes that were frequently selected for the models. These findings may again reflect the complex relationships among the variables that increase the difficulty of distinguishing between class boundaries [28,29,64]. The consistent identification of INO80 complex components as the predominant features in our models substantiates the biological significance of our study and underscores the efficacy of integrated machine learning.

Given the persistent selection of subunits from Remodeler complexes other than INO80, we examined the biological significance of these subunits. For example, Swr1, a protein from the SWR complex, was frequently selected. This may be attributed to its closer evolutionary relationship with INO80 components compared to other complex subunits such as Vps71, Vps72, or Arp6, as shown in Fig. 5C. This evolutionary connection between SWR1 and INO80, believed to have originated from a common ancestor, helps explain their structural similarities and potential functional overlap [59]. Therefore, the consistent selection of Swr1 by the models across both outcome datasets shows the cross-talk and interdependency between related chromatin remodeling complexes.

Our research establishes the effectiveness of machine learning techniques in network perturbation analysis to characterize structure-function relationships in protein complexes, demonstrating their broad applicability. Since our approach is based on quantitative measures with specified outcomes, it is adaptable to diverse biological systems beyond yeast. In principle, this model could extend to organisms such as mice, humans, and plants, provided that comparable quantitative data on protein interactions and complex structures are available. For predicting structural complexes in these systems, ensemble methods like Random Forests or Gradient Boosting might be particularly effective due to their robustness and ability to handle complex biological data [56,65]. However, the optimal model choice would depend on the specific characteristics of the available data.

In addition to its applicability to different biological systems, our integrative approach can be adapted to other types of perturbation data, including those involving mutations, inhibitors, or other interventions. This adaptability broadens the potential applications of these methods, including their extension across species. For instance, although our investigation focused exclusively on the four yeast chromatin remodeling complexes for the prediction of the yeast INO80 complex, the conservation of these complexes between yeast and human suggests that these data could serve as a training dataset for understanding perturbations in the human INO80 complex. Such applications could further validate and extend our research by enabling the characterization of structure-function relationships in more protein complexes and other species. Consequently, the implications could advance our understanding of disease mechanisms and drug development, significantly impacting fields such as biomedical science and clinical research.

While this adaptability presents exciting opportunities, it also introduces challenges when extending predictions across species that affect performance and interpretation. A key limitation is the evolutionary divergence among species, which results in differences in protein sequences and interaction networks. Predictors trained on one species, like yeast, may not work well for others, such as mammals, especially for proteins with low sequence conservation (<30 % identity) [66,67]. Data availability and quality are also major obstacles, as non-model organisms often lack comprehensive datasets, leading to biased models. Furthermore, variations in functional annotations and biological responses to perturbations complicate the establishment of equivalent feature sets across species. For example, a drug may behave differently in mouse models compared to humans due to species-specific metabolic and structural differences. To address these issues, future studies should perform species-specific parameter tuning and integrate structural constraints to improve model adaptability across biological systems.

Another limitation to our study lies in the validation of our predictions. While we employed k-fold cross-validation (5-fold for the first outcome, 8-fold for the second) to assess model performance and biological validation through phylogenetic relationships, the lack of experimental validation constrains the robustness of our findings. Internal validation provides insights into the predictive capability of our machine learning models within the context of our dataset; however, external validation through experimental approaches is necessary to confirm the biological relevance of the predicted interactions beyond the evolutionary connections observed. One strategy developed by Deng et al. [68] and Lu et al. [69] involved conducting knockout experiments to validate machine learning predictions, demonstrating the accuracy and broad applicability of their approach. Future work could adopt a similar strategy, such as perturbing Arp5 and assessing its impact on Yta7 interactions, to further validate our predictions and enhance the practical relevance of our integrative approach.

## 5. Conclusion

Protein networks exhibit significant complexity, characterized by numerous interactions, signaling pathways, and regulatory mechanisms. Perturbations within these networks, notably those induced by genetic mutations, can potentially induce substantial shifts in cellular behavior. Given their ability to discern patterns and relationships in extensive, multi-dimensional datasets that prove challenging for traditional analytical methods, machine learning models emerge as well-suited tools for navigating this intricate complexity. In our study, we demonstrated the effectiveness of an integrative machine learning approach in network perturbation research by showcasing its ability to predict perturbation patterns in the INO80 complex in *S. cerevisiae* while preserving the biological context of the data. By combining structural analysis with machine learning techniques, we captured and represented the perturbed protein interaction networks of the complex, which shed light on the structure-function relationship. Our findings highlight the robustness and potential of integrative methods in network analysis for understanding protein complex functionality. Future studies utilizing these techniques could further enhance our understanding of functional prediction in protein interaction networks and contribute to areas such as cross-species comparison, disease research, and drug development prediction outcomes.

## Supplementary Material

1

2

3

Supplementary data to this article can be found online at https://doi.org/10.1016/j.jprot.2025.105439.

## Figures and Tables

**Table 1:** Machine learning model performance evaluation of TopS normalized data when the outcome is presence vs. absence of proteins in at least one module in the perturbed INO80 network.

Metrics	DT	RF	XGBoost	ADABoost	Naive Bayes	LSVM	NLSVM	PSVM	LR	LDA	Lasso	Ridge
Accuracy	**0.82**	**0.89**	**0.83**	**0.88**	0.82	0.82	0.85	0.87	0.81	0.82	0.83	0.83
Sensitivity	**0.81**	**0.97**	**0.82**	**0.96**	0.99	0.99	0.98	0.99	0.98	0.98	0.95	0.96
Specificity	**0.87**	**0.54**	**0.85**	**0.54**	0.13	0.11	0.35	0.39	0.13	0.17	0.33	0.29
AUC	**0.84**	**0.75**	**0.83**	**0.75**	0.56	0.55	0.66	0.69	0.56	0.57	0.64	0.62

DT, decision tree; RF, random forest; LSVM, linear support vector machine; NLSVM, nonlinear support vector machine; PSVM, polynomial support vector machine; LR, logistic regression; LDA, linear discriminant analysis.

**Table 2:** Machine learning model performance evaluation of percentage-row normalized data when the outcome is significantly affected vs. not significantly affected proteins (z > 1.5 or z < −1.5) in the perturbed INO80 networks.

Metrics	DT	RF	XGBoost	ADABoost	Naive Bayes	LSVM	NLSVM	PSVM	LR	LDA	Lasso	Ridge
Accuracy	0.616	0.628	**0.638**	**0.645**	0.576	0.628	0.626	0.635	0.638	0.631	0.635	0.631
Sensitivity	0.275	0.451	**0.549**	**0.412**	0.511	0.264	0.159	0.39	0.33	0.346	0.324	0.264
Specificity	0.872	0.761	**0.704**	**0.819**	0.626	0.901	0.975	0.819	0.868	0.844	0.868	0.905
AUC	0.574	0.606	**0.627**	**0.616**	0.568	0.582	0.567	0.605	0.599	0.595	0.596	0.585

DT, decision tree; RF, random forest; LSVM, linear support vector machine; NLSVM, nonlinear support vector machine; PSVM, polynomial support vector machine; LR, logistic regression; LDA, linear discriminant analysis.

## Data Availability

The mass spectrometry datasets are publicly available in [3, 16] and reported in the supplementary Tables S1 and S2.
