# Supplementary figures and images for: Integrative approaches for predicting protein network perturbations through machine learning and structural characterization
Source: J Proteomics. Author manuscript; Available in PMC 2026 May 18. (PMC13181614; doi:10.1016/j.jprot.2025.105439)

## Ino80 TAP - *nph10*Δ

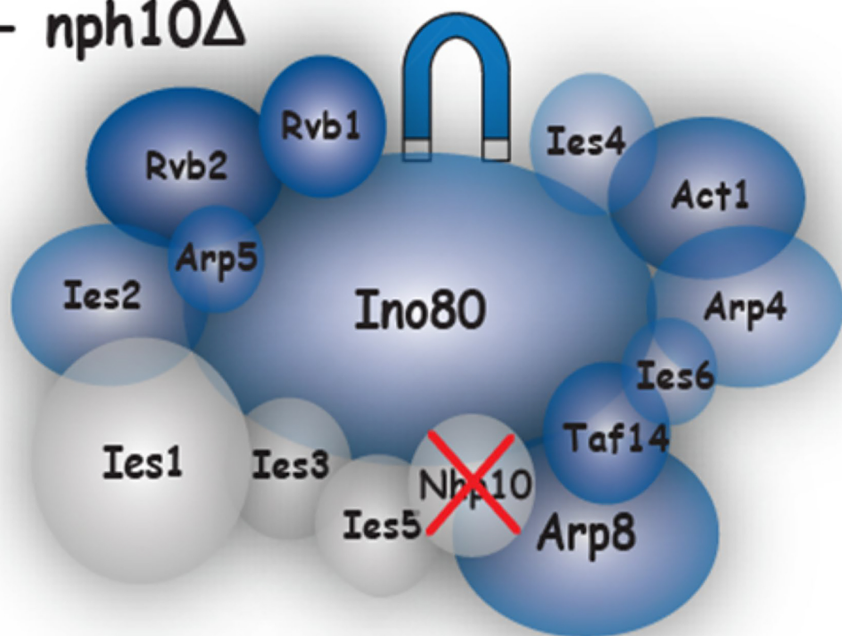

Supplement: 1 [file NIHMS2174446-supplement-1.pdf]
